# VertXNet: an ensemble method for vertebral body segmentation and identification from cervical and lumbar spinal X-rays

**DOI:** 10.1038/s41598-023-49923-3

**Published:** 2024-02-09

**Authors:** Yao Chen, Yuanhan Mo, Aimee Readie, Gregory Ligozio, Indrajeet Mandal, Faiz Jabbar, Thibaud Coroller, Bartłomiej W. Papież

**Affiliations:** 1 Novartis Pharmaceuticals Corporation, East Hanover, NJ USA; 2 Big Data Institute, University of Oxford, Oxford, UK; 3 John Radcliffe Hospital, Oxford University Hospitals NHS Foundation Trust, Oxford, UK

**Keywords:** Radiography, Computer science

## Abstract

Accurate annotation of vertebral bodies is crucial for automating the analysis of spinal X-ray images. However, manual annotation of these structures is a laborious and costly process due to their complex nature, including small sizes and varying shapes. To address this challenge and expedite the annotation process, we propose an ensemble pipeline called VertXNet. This pipeline currently combines two segmentation mechanisms, semantic segmentation using U-Net, and instance segmentation using Mask R-CNN, to automatically segment and label vertebral bodies in lateral cervical and lumbar spinal X-ray images. VertXNet enhances its effectiveness by adopting a rule-based strategy (termed the ensemble rule) for effectively combining segmentation outcomes from U-Net and Mask R-CNN. It determines vertebral body labels by recognizing specific reference vertebral instances, such as cervical vertebra 2 (‘C2’) in cervical spine X-rays and sacral vertebra 1 (‘S1’) in lumbar spine X-rays. Those references are commonly relatively easy to identify at the edge of the spine. To assess the performance of our proposed pipeline, we conducted evaluations on three spinal X-ray datasets, including two in-house datasets and one publicly available dataset. The ground truth annotations were provided by radiologists for comparison. Our experimental results have shown that the proposed pipeline outperformed two state-of-the-art (SOTA) segmentation models on our test dataset with a mean Dice of 0.90, vs. a mean Dice of 0.73 for Mask R-CNN and 0.72 for U-Net. We also demonstrated that VertXNet is a modular pipeline that enables using other SOTA model, like nnU-Net to further improve its performance. Furthermore, to evaluate the generalization ability of VertXNet on spinal X-rays, we directly tested the pre-trained pipeline on two additional datasets. A consistently strong performance was observed, with mean Dice coefficients of 0.89 and 0.88, respectively. In summary, VertXNet demonstrated significantly improved performance in vertebral body segmentation and labeling for spinal X-ray imaging. Its robustness and generalization were presented through the evaluation of both in-house clinical trial data and publicly available datasets.

## Introduction

**Figure 1:**
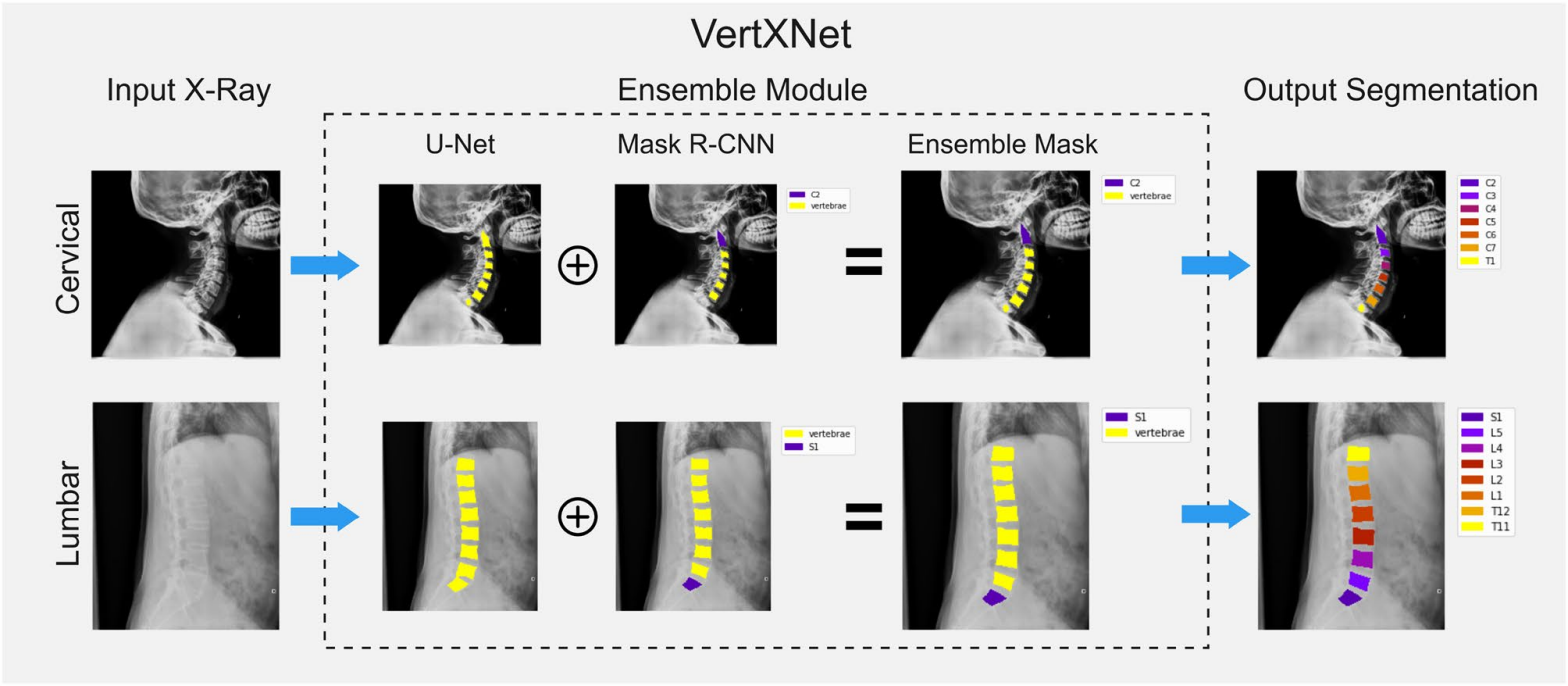
VertXNet overview: (1) An ensemble method (U-Net and Mask R-CNN) produces a robust segmentation for each vertebral body, (2) Mask R-CNN locates a reference vertebral body (either C2 or S1) and infers remaining vertebral bodies.

X-ray imaging is a fast and low-cost modality making it widely applied for spinal disease assessment and monitoring. Reliable vertebral body annotation (i.e., vertebral body segmentation and identification) from spinal X-ray images is a prerequisite to quantitatively perform functional analysis of the spine using machine learning-based methods, e.g., surgical planning or abnormality quantification^1^. Many image segmentation and detection methods proposed in the last decade have been proven to be applicable for biomedical image analysis such as U-Net^2^ and Mask R-CNN^3^. However, there are still challenges in translating those tools into specific medical imaging tasks. As opposed to natural images, medical images (including spinal X-rays) have relatively limited spatial resolution. X-rays in particular are single-plane images (compared to Magnetic Resonance Imaging (MRI), which is 3D imaging) that often exhibit a lower signal-to-noise ratio. A unique challenge for vertebral body annotation in lateral spinal X-ray images is differentiating one vertebral body from another vertebral body as neighboring vertebral bodies would share similar shape and intensity (e.g. see the exemplar images in Fig. 1). Data scarcity, commonly seen in medical applications, furthermore constrains the applicability of deep learning methods for the task, as a large amount of annotated training data is required for such methods to produce accurate results. This is because manual annotation of X-ray images is non-trivial and must be performed by experts, which is time-consuming and expensive, but also prone to errors (i.e., inter- and intra-reader variability). Therefore, a solution that could automatically segment and identify the vertebral body in the spinal X-ray images would greatly decrease the cost, time, and inter-observer errors caused by human experts, and thus foster analysis of large X-ray imaging repositories.

### Our contribution

To address these problems, we proposed a novel ensemble method VertXNet that combined two SOTA segmentation and detection networks, namely **U-Net** and **Mask R-CNN**. By combining the two networks, we can not only benefit from the advantages of semantic (U-Net) and instance (Mask R-CNN) segmentation networks in a single approach to produce robust segmentation results, but also the identities of the vertebral body can be accurately inferred in the final output according to the detection of the reference vertebral body.

The preliminary results of VertXNet were presented as a conference publication^4^ and this paper aims to extend the previous work as follows. We provide detailed descriptions of our pipeline, including the key rule-based scheme that robustly combines segmentation outputs from both U-Net and Mask R-CNN. In order to demonstrate the generalizability on other spinal X-rays, we tested our pipeline using datasets acquired from multiple centers and demonstrated that our pipeline is robust due to the ensemble architecture. Finally, we also create extra labels (i.e., landmarks for the reference vertebral body) that are missing in the publicly available NHANES II dataset to enable the proposed pipeline to be compared to other methods. To summarize:

1. In contrast to a conventional end-to-end deep-learning approach, our pipeline combined two SOTA networks (i.e. U-Net and Mask R-CNN) leveraging the advantages from both semantic and instance segmentation and showing a better performance than each SOTA network alone.
2. Using a reference vertebral body to infer the identities of the remaining vertebral body according to their anatomical structure in X-ray images has shown greater performance and more robust prediction than predicting all vertebral bodies at once.
3. The segmentation and identification results of our pipeline were robust i.e., the proposed pipeline has been tested on 3 different datasets including two in-house datasets from different clinical trials and one public dataset with the reference vertebral bodies annotated by our internal radiologists.
4. Reference vertebral bodies of a public dataset (NHANES II) were annotated by our internal radiologists. The extra labels of reference vertebral bodies will be publicly shared alongside this investigation in order to replicate our results and encourage other teams to advance the field of spinal segmentation and identification (The annotation for reference vertebral bodies of NHANES II is given in “Data availability” section).

### Related work

Image segmentation is an attractive task thanks to the availability of larger datasets. Before the booming development of deep learning, medical image segmentation methods utilized hand-crafted features and rule-based models for producing a classification of each independent pixel^5^. Although the traditional deformable models, e.g. Active Contour Models^6^ (i.e., ACMs or so called ‘Snakes’), have proven to be effective methods that deform the contours towards the imaged object boundaries, recently convolutional neural networks (CNNs) have become the most promising method for segmentation, from natural to medical images^2,7,8^. Rashid et al.^9^ applied a fully convolutional neural network on segmenting lungs from chest X-rays. Novikov et al.^10^ also applied the convolutional neural networks for segmenting lungs, clavicles, and heart from chest X-rays. They demonstrated that the proposed pipeline was able to outperform the SOTA methods by combining a series of techniques such as delayed sub-sampling, exponential linear units, and highly restrictive regularization.

Many studies have also been carried out for fully automatic vertebral body segmentation and identification in spinal X-ray images. Arif et al.^11^ developed a network to predict shapes instead of performing pixel-wise classification for vertebral body segmentation in X-ray images. Tran et al.^12^ proposed the MBNet which was a multi-task deep neural network for semantic segmentation on lumbar vertebral bodies. Li et al.^13^ proposed a CNN model that combined two types of features of lumbar vertebral body X-ray images to automatically detect lumbar vertebral bodies for C-arm X-ray images. Kurachka et al.^14^ proposed to use a CNN based method trained on patches from spinal X-ray images for vertebral bodies detection. The model produced the likelihood of a vertebral body being contained by the given patch. Li et al.^15^ proposed a novel and computation-efficient network, called SPA-ResUNet, which combined residual U-Net with strip-pooling attention mechanism for the multi-class vertebral bodies and inter-vertebral discs segmentation. Whitehead et al.^16^ proposed a coarse-to-fine pipeline for spinal segmentation which used a series of four pixel-wise segmentation networks to refine the segmentation results step by step. Bidur et al.^17^ proposed an automatic deep learning-based method that firstly detects the vertebral bodies and then predicts 4 landmark corners for each vertebral body to finally produce the Cobb Angles for spinal X-ray images. Ruhan et al.^18^ fine-tuned a pre-trained faster R-CNN on a small annotated X-ray dataset, and they demonstrated that the faster R-CNN can outperform the traditional sliding window detection method with hand-crafted features. Kim et al.^19^ proposed multi-dilated recurrent residual U-Net (MDR2-UNet) to perform vertebral body segmentation such that the vertebral compression ratio can be accurately measured. Winsor et al.^20^ proposed a novel approach for labelling vertebral body sequences based on language modelling. The proposed approach reached SOTA performance over a range of clinical datasets. Cho et al.^21^ developed a fully automated pipeline based on a U-Net and for assisted evaluation of lumbar lordosis. Zhang et al.^22^ fine-tuned a pre-trained Mask R-CNN to predict the vertebra level(s) on the sagittal X-rays taken by smartphones or screenshots. Shin et al.^23^ also utilized a U-Net to segment vertebral bodies for analyzing the temporal trends in cervical curvature.

There are also many studies of vertebral body segmentation based on CT (Computerised Tomography) that have been investigated in recent years. Qadri et al.^24^ proposed OP-convNet, which is an overlapping patch-based model, for automatic vertebrae CT image segmentation. They employed overlapping patches in segmentation tasks using 2D convNet in order to reduce memory usage and the risk of overfitting. Altini et al.^25^ proposed a framework for vertebral body segmentation and identification from CT images combining both deep learning and classical machine learning methodologies. The proposed method consists two steps: a binary fully automated segmentation of the whole spine, using a 3D CNN, and a semi-automated procedure using traditional machine learning algorithms for locating vertebral bodies’ centroids. Cheng et al.^26^ proposed to use of a 2-step deep-learning approach for automatic CT vertebrae localization and segmentation. The first step used a U-Net to localize vertebral body centroids. The second step segmented the specific vertebral body within a region of interest determined by the centroids obtained from the first step.

The studies mentioned above have achieved remarkable performance in the vertebral body segmentation task. However, most methods still use either semantic segmentation models (e.g. U-Net) or instance segmentation models (e.g. Faster R-CNN, Mask R-CNN) as the backbones, which cannot take advantage of both approaches. By combining two segmentation paradigms and utilizing the proposed ensemble approach, we can mitigate the weaknesses of individual models, enhance their generalization ability, and improve overall performance.

## Methodology

The proposed pipeline, VertXNet, is trained and tested on either anonymized, in-house X-ray images (MEASURE 1 and PREVENT) from completed and anonymized secukinumab (a fully human anti-IL-17A monoclonal antibody) axSpA (axial spondyloarthritis) clinical trials^27^ or the digitized version of the X-ray films from NHANES II dataset. As shown in Fig. 1, VertXNet employed a 2-steps pipeline, *segmentation* and *identification* of spinal vertebral bodies. X-ray images were fed into a segmentation module to first generate segmentation mask of each vertebral body. Two SOTA segmentation networks of different mechanisms, U-Net^3^ and Mask R-CNN^3^, have been investigated. U-Net performs semantic segmentation which classifies each pixel of the image, and Mask R-CNN performs instance segmentation which segment instance within bounding boxes. However, according to our preliminary results, neither of them worked perfectly individually. Thus, the two networks are trained independently in order to produce their own predictions. Then, a rule-based ensemble method is introduced to combine outputs from both networks to robustly produce both vertebral bodies segmentation and identification. From there, we inferred the identifications of the rest of the vertebral bodies starting from the detected reference vertebral bodies (one per image).

### U-Net

**Figure 2:**
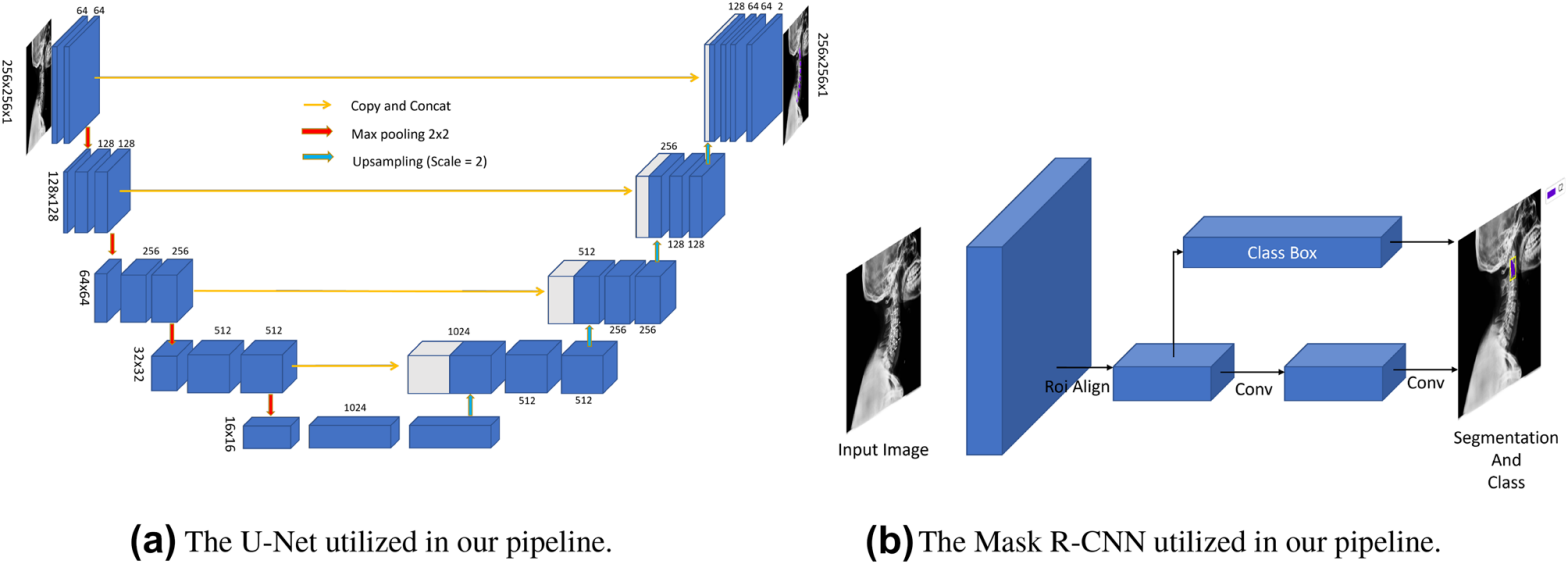
Two segmentation models used in our pipeline, namely U-Net and Mask R-CNN.

Our pipeline uses a modified version of the original U-Net as illustrated in Fig. 2a for semantic segmentation. The downscale path has 5 convolutional blocks, each composed of two convolutional layers with a filter size of $3\times 3$, stride of 1, padding of 1 in both directions and followed by batch normalization and ReLU (Rectified Linear Unit) activation. Max pooling with a kernel size of 2 is applied at the end of each block. At the bottleneck, the number of feature maps increases from 1 to 1024. In the upscale path, every block starts with a upsample layer with a scale factor of 2, which doubles the size of feature maps in both directions but decreases the number of feature maps by two. Two convolutional layers of filter size 3x3 and padding of 1 are applied on the concatenation of upsample feature maps and the feature maps from the encoding path followed by batch normalization and ReLU activation. Max pooling of kernel size 2 is used at the end of each block. The last layer is a $1\times 1$ convolutional layer to collapse the number of features and predict our foreground/background classes (binary segmentation).

In order to demonstrate the flexibility of the proposed pipeline, we also replaced the U-Net with a 2D nnU-Net^28^ for semantic segmentation which is able to automatically configure itself to the task. We used the official release of the nnU-Net (https://github.com/MIC-DKFZ/nnUNet/tree/nnunetv1) in our pipeline and the pipeline with nnU-Net yields better performance compared to the baseline U-Net.

### Mask R-CNN

In our pipeline, Mask R-CNN not only detects the objects in an image but also simultaneously generates a precise segmentation mask for each instance (i.e., vertebral bodies). Mask R-CNN is an extension of Faster R-CNN^29^ which has two predictions for each candidate object, a category, and an offset for the bounding box. Mask R-CNN adds a third branch that produces the object mask as an additional output besides the bounding box and its category (Fig. 2b). The additional output of the segmentation mask is different from the category and bounding box outputs, requiring the extraction of a much finer spatial layout of an object. In our pipeline, we used the pre-trained components provided in PyTorch library^30^ for performing the instance segmentation. A pre-trained ResNet-50 with Feature Pyramid Network (FPN) is used as the backbone of our Mask R-CNN.

### Ensemble rules

**Figure 3:**
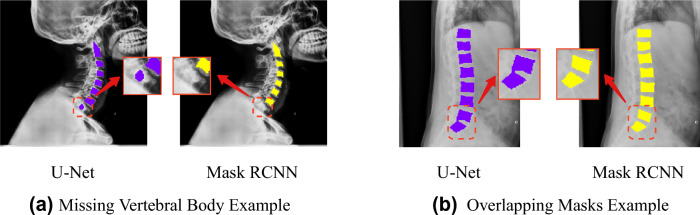
Exemplar failure cases on mask prediction with individual SOTA method; (**a**) Mask R-CNN fails to detect vertebral body T1 due to the incomplete of shape, however, U-Net successfully annotates the partial shape of vertebral body T1; (**b**) U-Net generates overlapping contours and cannot be easily separated, however, Mask R-CNN generates instance segmentation within bounding box which makes all vertebral bodies separable.

**Figure 4:**
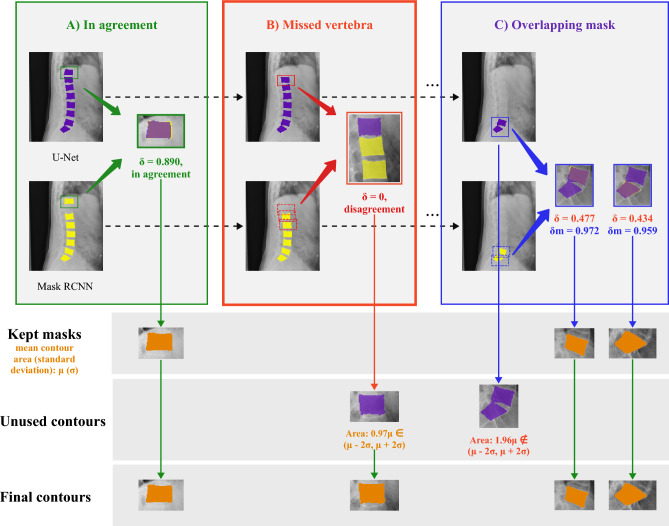
VertXNet ensemble procedure: Pairwise comparison between two segmentation results are performed; (**A**) if two masks are in agreement, the union of two masks will be added to the list of ensemble masks, and the original masks will be removed from each segmentation; (**B**) if the mask cannot find the mask in agreement/partial agreement from the other segmentation, it will be added to the unused contours for further check; (**C**) if Mask R-CNN segmentation overlapped by a large proportion with U-Net but not the other way around, the Mask R-CNN generated mask will be added to the list of ensemble masks. At the end of each procedure, the mean and standard deviation of ensemble masks will be calculated and valid masks in unused contours with similar size will be added to the ensemble masks list.

In our preliminary experiments, neither of these two independent methods (U-Net and Mask R-CNN) could perfectly solve the vertebral body segmentation task (Fig. 3). Previous works^31,32^ also showed the limitation of the single methods and required pre-processing of the medical image, modification of framework, or incorporation of prior knowledge. We noticed that Mask R-CNN may have missed the prediction for those vertebral bodies, which have the incomplete boundary since the first step of object detection fails to identify the bounding box of the incomplete vertebral body, while U-Net is more reliable to find vertebral bodies as it performs semantic segmentation, that is binary classification at pixel level (Fig. 3a). However, under the setting of binary classification, U-Net may generate overlapping masks when two vertebral bodies in the X-ray image are located closely to each other due to pose or illness, like vertebral unit space narrowing or growing of syndesmophytes. Moreover, unlike Mask R-CNN performing instance segmentation within the bounding box, it is challenging for U-Net to separate the overlapping masks as illustrated in Fig. 3b. Therefore, the proposed pipeline would jointly consider the outputs from both U-Net and Mask R-CNN in order to address the challenges faced by each individual model (see Algorithm 1 and Fig. 4).

#### Selected candidate masks in agreement

 In the ensemble method, we first obtain output masks from both U-Net and Mask R-CNN. Instance contours can be easily extracted from Mask R-CNN given predicted bounding boxes. We apply the contour approximation method on the semantic segmentation to extract the instance contours from U-Net. U-Net contours are denoted as $\{C^{u}_1,..., C^{u}_m\}$ and Mask R-CNN contours $\{C^{m}_1,..., C^{m}_n\}$. Then we define the agreement between two contours by

$$\begin{aligned} \delta (C_i, C_j) = min\left( \dfrac{area(C_i \cap C_j)}{area(C_i)}, \dfrac{area(C_i \cap C_j)}{area(C_j)}\right) = \dfrac{area(C_i \cap C_j)}{max(area(C_i), area(C_j))} > \eta \end{aligned}$$

$\eta$ is a pre-defined threshold value, and we recommend 0.6 as the cutoff. The choice can be flexible within a range, in our experiment, $\eta$ from 0.3 to 0.7 generated similar results. Then a cross-comparison between the contours of U-Net and contours of Mask R-CNN, i.e. $\{(C^{u}_i, C^{m}_j)|$ for all $i \in 1,..., n$ and $j \in 1, ..., m\}$, is performed to select the candidate vertebral body masks. If the a mask contour from U-Net and Mask R-CNN is selected as in agreement, the union of these pair of contours will be kept in the final list of vertebral masks.

#### Separate overlapping masks

After the first step, not all candidate masks have been selected. Neither of the missed masks and the overlapping masks from the scenario we demonstrated in Fig. 3 will be selected as candidate masks. We first targeted on overlapping masks. Overlapping masks happen when two vertebral bodies are too close to each other or overlap on the X-ray. In such case, the contour approximation will not be able to split such two vertebral bodies given semantic segmentation from U-Net. However, Mask R-CNN provides instance segmentation with bounding box, the instance masks are always separable. We check again the intersection of two contours given by U-Net and Mask R-CNN. If the intersection occupies a good percentage, e.g., $\eta$ defined in the first step, of Mask R-CNN contours, but relatively small on U-Net contours, it is very likely that this is an overlapping mask on U-Net. Here we denote $\delta _m(C^u_i, C^m_j) = \dfrac{area(C^u_i \cap C^m_j)}{area(C^m_j)}$ and use criteria $\delta < \eta$ and $\delta _m > \eta$ to define the finding of overlapping mask. In such case, we can directly take instance contours from Mask R-CNN as the candidate masks.

#### Pick up missed vertebral bodies

 Finally, we have one more challenge to tackle, missed vertebral bodies by one method. In order to pick up potential missed vertebral bodies, all contours that have not been selected as valid candidate masks will be further investigated comparing with kept masks. The area of the kept masks will be measured, the mean $\mu _C$ and standard deviation $\sigma _C$ on the list of kept masks will be calculated for the evaluation of the unused contours. The unused contours whose area are within $\lambda \cdot \sigma _C$ range of $\mu _C$ will be picked up as missed valid final contours. The $\lambda$ is a hyper-parameter to be selected. The summary statistics from kept contours provide additional justification for the missed vertebral bodies and reduce the risk of false negative by dropping all unused contours or false positive by adopting all unused contours.

#### Sort all candidate masks

 After all three steps, we have a complete set of vertebral body masks that have worked around the issues faced by either individual method. The selected instance masks are sorted vertically on the image for the next step of inferring and labelling.

**Algorithm 1:**
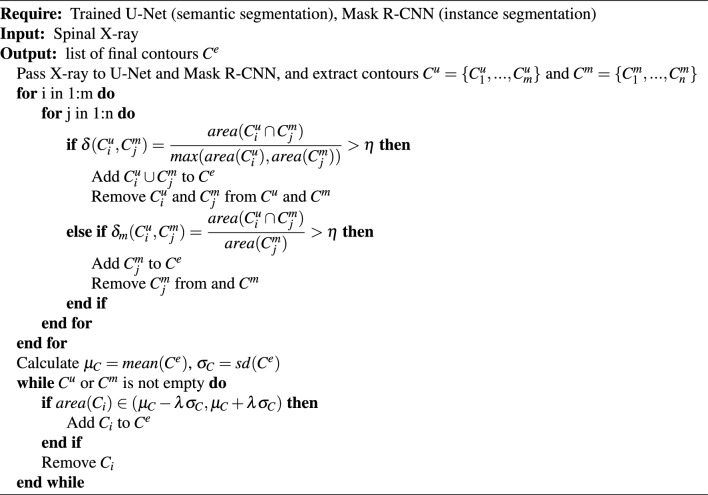
Pseudo code for vertebral bodies ensemble rules.

### Vertebral bodies identification

Due to the similarity in shape, it is challenging to correctly label the vertebral body under multi-class task settings (see Table 1). The spatial relationship among vertebral bodies, such as the knowledge that L1 (lumbar #1 vertebral body) should be followed by L2 (lumbar #2 vertebral body), can be used. Thus finding the reference vertebral bodies becomes essential to properly label all remaining vertebral bodies in an X-ray image. Luckily, both lumbar and cervical images contain two types of vertebral bodies that can be easily distinguished from others, and are identified as the reference vertebral bodies. The reference vertebral bodies include: (1) ‘C2’, cone-shaped and the first detectable vertebral body at the top of the cervical X-ray image, and (2) ‘S1’, a triangular-shaped vertebral body, which is commonly the last visible vertebral body at the bottom of a lumbar X-ray. To detect the reference vertebral bodies, the Mask R-CNN is trained to distinguish the reference vertebral body from other vertebral bodies. If C2 is detected on an image, we simply “zip” down the spine to infer the cervical (C) and thoracic (T) labels (from C3 to C7 and T1). On the other hand, if ‘S1’ is detected we “zip” up the spinal vertebral bodies to infer the lumbar (L) and thoracic (T) labels (from L5 to L1, and T12 to T11).

## Experiments and results

### Datasets

The proposed pipeline was developed and evaluated on three spinal X-ray datasets. Two of the anonymized datasets were obtained from the secukinumab axial spondyloarthritis global clinical trials, MEASURE 1^27^ and PREVENT^33^, and the third was from a public dataset of spinal X-rays (i.e., NHANES II).

#### NHANES II

During the Second National Health and Nutrition Examination Surveys (NHANES II), 17,000 lateral cervical/lumbar X-ray films were collected to provide evidence of osteoarthritis and degenerative disc disease. The NHANES II dataset also provided landmark annotation for vertebral bodies in 544 images, but only C3–C7 and L1–L5 were provided with complete landmark coordinates to generate masks. Our internal radiologists annotated the reference vertebral bodies (i.e., C2 and S1) when they were visible in the dataset. Eventually, we got 445 out of 544 images with reference vertebral bodies. However, the quality of the X-ray film in NHANES II is lower than in our in-house clinical datasets.

#### MEASURE 1

A sample of images from the anonymized MEASURE 1 study^27^ were utilized, in which sagittal cervical and lumbar X-ray images were acquired at different visits (Baseline, Week 104, and Week 208). All vertebral bodies in the 512 X-ray images (293 cervical and 219 lumbar) were annotated by our internal radiologist following the annotation procedure of NHANES II. During the annotation, our internal radiologist assigned 8 landmarks (same as the annotation process for NHANES II) and their identities (e.g. C2, C3 .... ) to each vertebral body in the X-ray images.

#### PREVENT

Another anonymized in-house clinical trial dataset with a different population of axSpA (axial spondyloarthritis) patients, PREVENT^33^ was also investigated. Visible vertebral bodies from 226 lateral X-rays (132 cervical and 94 lumbar) were annotated by the same internal radiologists. Compared with MEASURE 1, the X-ray films in PREVENT captured a slightly wider field of view of the spinal anatomy, which meant more T1, T11 and T12 were visible in the PREVENT dataset. The manual annotation process of PREVENT follows the same protocol as MEASURE 1, where 8 landmarks and the identity of a vertebral body is provided by our radiologist

### Experiments

#### Ensemble vs individual methods

 The 512 annotated X-rays from MEASURE 1 were randomly split and stratified by spinal acquisition type. 80% of the X-rays images were used for training the pipeline and the remaining 20% for testing. The two models (U-Net and Mask R-CNN) were trained on the training dataset and the Dice coefficients were calculated on the testing set to compare the two models. Moreover, we also applied a 2D nnU-Net on our task in order to carry out a comprehensive comparison. Our pipeline’s mean Dice coefficient was **0.90**, compared with Mask R-CNN’s **0.73**, U-Net’s **0.71**. Detailed Dice coefficients on each vertebral type have been provided in Table 1. A drop in performances across models was observed for T1, T11, and T12 due to data imbalance caused by the lack of appearance of these vertebral bodies in X-rays. Greater performance from our model was observed for T1, T11, and T12. We also investigated the occurrence of each scenario in our algorithm, i.e. contours in agreement, overlapping mask, and missed vertebral bodies. We extracted 635 vertebral bodies in total from 108 X-rays in test set, among which there were 519 contours in agreement between U-Net and Mask R-CNN, 69 missed vertebral bodies and 47 extracted from overlapping masks. This suggests that on average at least one mechanism will encounter some issue on each individual X-ray, and the ensemble masks not only solved the potential risk of overlapping masks or missed vertebral body masks by one model but also improved the Dice coefficients of the outputs. Besides U-Net, we also investigated the most recent SOTA semantic segmentation method nnU-Net and compared the results with ours. nnU-Net performs comparable results with our pipeline, but it is much more computationally expensive and still does not solve the issue we mentioned for semantic segmentation. Since our pipeline fuses two models from different mechanisms, nnU-Net can be used to replace U-Net as the SOTA model for semantic segmentation. We implemented VertXNet-v2 with nnU-Net and have achieved better performance. The detailed Dice coefficients are provided in Table 1 and the results in the rest of the paper are performed on VertXNet-v2 which combined nnU-Net and Mask R-CNN.

**Table 1:** Model comparison (numbers in bold indicate the best performance).

(a) Dice coefficients of cervical vertebral bodies on test set								
Model	Overall	C2	C3	C4	C5	C6	C7	T1
Mask R-CNN	0.730	0.852	0.882	0.889	0.842	0.767	0.736	0.325
U-Net	0.712	0.823	0.846	0.848	0.822	0.809	0.717	0.067
nnU-Net	0.902	0.912	**0.922**	0.902	0.881	0.872	0.788	0.392
**VertXNet**	0.899	0.905	0.912	0.912	0.906	0.901	0.881	0.518
**VertXNet-v2**	**0.912**	**0.917**	**0.922**	**0.923**	**0.916**	**0.917**	**0.903**	**0.542**

(b) Dice coefficients of lumbar vertebral bodies on test set								
Model	T11	T12	L1	L2	L3	L4	L5	S1
Mask R-CNN	0.379	0.577	0.706	0.730	0.615	0.707	0.732	0.744
U-Net	0.338	0.519	0.643	0.726	0.785	0.772	0.726	0.603
nnU-Net	0.684	0.795	0.827	0.880	0.892	0.891	0.882	0.784
**VertXNet**	**0.761**	0.843	0.864	0.870	0.871	0.865	0.854	0.823
**VertXNet-v2**	0.716	**0.858**	**0.885**	**0.899**	**0.900**	**0.896**	**0.888**	**0.832**

The performance on the test set between VertXNet and the three benchmark models was demonstrated in this table. VertXNet-v2 surpassed benchmark models in nearly all vertebral body categories.

#### Generalizability of pre-trained pipeline

**Figure 5:**
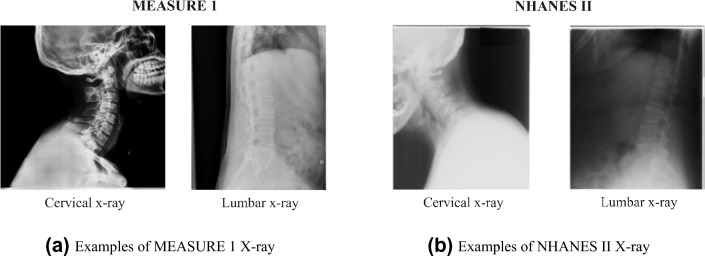
Examples of X-rays from clinical trial and public dataset; MEASURE 1 X-rays have better quality in terms of resolution, contrast, etc. in comparing with NHANES II. Quality of X-rays are similar in PREVENT and MEASURE 1.

**Figure 6:**
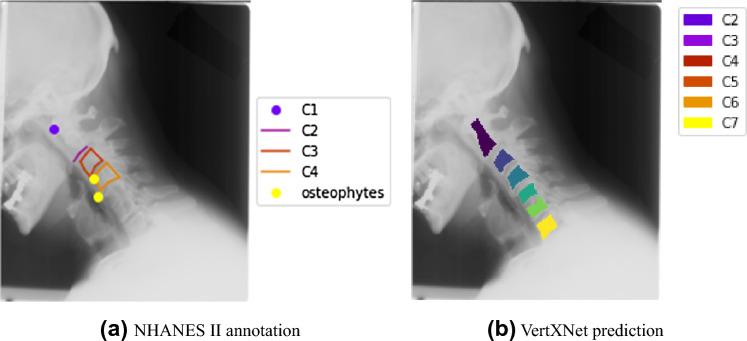
One of the reasons that the evaluation metrics (i.e., Dice coefficients and Panoptic quality score) dropped on the NHANES II dataset was due to some incomplete annotations on NHANES II that have made the evaluation unfair when they were treated as ground truth. Figure 6a shows an example of NHANES II annotation with only a lower edge of C2, C3, and C4, but in Fig. 6b, VertXNet has correctly segmented and labeled C2–C7.

 The pre-trained pipeline on the MEASURE 1 dataset was also tested on PREVENT and NHANES II to evaluate the generalization performance on other spinal X-ray datasets. The Dice coefficients are reported in Table 2. The overall Dice coefficients for the proposed method are similar among the two datasets, with **0.90** on PREVENT and **0.88** on NHANES II. A boost of performance on T1 of PREVENT was maybe seen due to the wider visibility range on the films, similarly for T11. NHANES II experienced some drop in performance mainly due to two reasons, different image quality (see Fig. 5) and missing annotation on some vertebral bodies (see Fig. 6).

**Table 2:** Generalization of pre-trained pipeline on other datasets.

(a) Dice coefficients of cervical vertebral bodies on test set								
Dataset	Overall	C2	C3	C4	C5	C6	C7	T1
PREVENT (VertXNet-v2)	0.900	0.913	0.915	0.910	0.889	0.874	0.883	0.831
NHANES II (VertXNet-v2)	0.880	0.894	0.888	0.871	0.849	0.818	0.615	–

(b) Dice coefficients of lumbar vertebral bodies on test set								
Dataset	T11	T12	L1	L2	L3	L4	L5	S1
PREVENT (VertXNet-v2)	0.810	0.845	0.845	0.850	0.856	0.856	0.903	0.857
NHANES II (VertXNet-v2)	–	–	0.834	0.852	0.853	0.856	0.901	0.883

The pre-trained pipeline on MEASURE 1 has been tested on PREVENT and NHANES II to show the generalization capability of VertXNet-v2. The pre-trained pipeline has demonstrated consistent results on datasets with different patient populations (PREVENT) or different image quality (NHANES II) when comparing with MEASURE 1.

In some cases, (for example in Fig. 6) there is no annotation for C3-C7 provided in NHANES II, but our pipeline can correctly detected those vertebral bodies. Such cases can cause the Dice for individual vertebral body dropping to 0 since the intersection of predicted masks and ground truth is empty. The panoptic quality^34^ is also provided for the comparison in Table 3 in order to demonstrate the overall segmentation and identification performance of the proposed pipeline.

**Table 3:** Panoptic quality.

(a) Panoptic quality of cervical vertebral bodies on test set									
Dataset	Thresholding value	C2	C3	C4	C5	C6	C7	T1	
MEASURE 1	0.8	0.780	0.869	0.870	0.859	0.844	0.797	0.408	
	0.7	0.849	0.895	0.884	0.873	0.887	0.830	0.408	
PREVENT	0.8	0.846	0.891	0.883	0.881	0.840	0.851	0.688	
	0.7	0.863	0.891	0.892	0.881	0.870	0.885	0.688	
NHANES II	0.8	0.592	0.827	0.824	0.768	0.772	0.703	–	
	0.7	0.730	0.872	0.861	0.826	0.817	0.725	–	

(b) Panoptic quality of lumbar vertebral bodies on test set									
Dataset	Thresholding value	T11	T12	L1	L2	L3	L4	L5	S1
MEASURE 1	0.8	0.796	0.855	0.883	0.915	0.893	0.886	0.850	0.713
	0.7	0.796	0.880	0.883	0.915	0.916	0.908	0.893	0.782
PREVENT	0.8	0.844	0.890	0.885	0.909	0.917	0.926	0.884	0.720
	0.7	0.865	0.897	0.900	0.909	0.925	0.926	0.899	0.808
NHANES II	0.8	–	–	0.733	0.828	0.821	0.845	0.834	0.590
	0.7	–	–	0.837	0.876	0.875	0.883	0.890	0.771

Panoptic quality was utilized to further demonstrate the robustness of the performance of VertXNet-v2 on all three datasets. The performance on both MEASURE 1 and PREVENT was good and similar with each other, performance on NHANES II dropped slightly due to the low quality of the images and ground truth.

The panoptic quality (PQ) is defined in Equation 1, where *p* and *g* denote the predicted mask and the ground truth masks, respectively. *TP*, *FP*, and *FN* denote true positive, false positive, and false negative. The top of the equation aims to sum all the Intersection Over Union (IoU) ratios among all the true positives (TP) pairs. The bottom of the equation can be considered as a weighted sum between precision and recall. Equation 2 gives a more intuitive form to understand this measurement. The left side is evaluating the segmentation quality, namely how good is the predicted segmentation mask compared to its ground truth. The right side is evaluating the recognition quality, namely how good vertebral body identification of the proposed pipeline is.

1 $$\begin{aligned} PQ&= \frac{\sum _{(p,g) \in TP} IoU (p,g)}{\mid TP \mid + 1/2 \mid FP \mid + 1/2\mid FN \mid } \end{aligned}$$

2 $$\begin{aligned} PQ&= \frac{\sum _{(p,g) \in TP} IoU (p,g)}{\mid TP \mid } \times \frac{\mid TP \mid }{\mid TP \mid + 1/2 \mid FP \mid + 1/2\mid FN \mid } \end{aligned}$$

## Discussion

Annotating vertebral bodies from spinal X-ray images is a crucial step to further investigate clinical diagnosis prediction (i.e. disease status, treatment response). Despite a large number of studies of medical image segmentation in the literature^5,35^, there are still aforementioned challenges in developing an automatic and robust pipeline with high performance using limited training samples.

**Figure 7:**
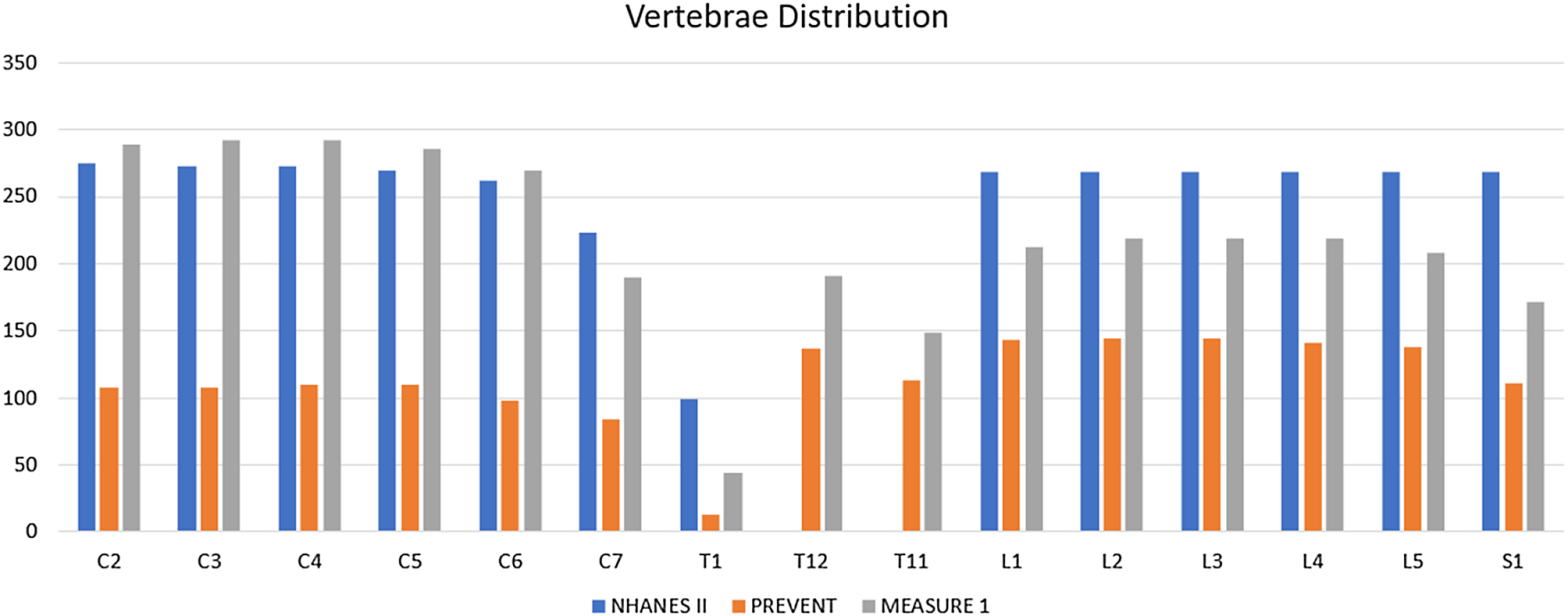
Vertebrae distribution of three datasets: NHANES II, PREVENT and MEASURE 1.

In this paper, we introduced an ensemble pipeline (VertXNet) to automatically segment and annotate vertebral bodies from spinal X-rays (working on either lumbar or cervical). VertXNet combines two segmentation mechanisms (i.e., semantic and instance segmentation) using a rule-based method, and demonstrated strong performances in our test sets. We evaluated our pipeline on three different datasets, namely MEASURE1, PREVENT and NHANES II, where two of them were from secukinumab clinical trials and the latter is publicly available annotated to fit the experiments. We demonstrated that the ensemble method significantly outperformed the U-Net and Mask R-CNN respectively using an unseen portion of our internal datasets. Great and robust performance was also observed on the different internal datasets and public dataset with different patient populations using the pre-trained pipeline.

Despite the promising results of our pipeline, there are still some limitations: (1) our pipeline used U-Net/nnU-Net and Mask R-CNN to produce segmentation masks for vertebral bodies independently. Our ensemble rule was then used to merge the segmentation masks to produce the final results. The ensemble rule was manually crafted and could have potential issues to not generalize well to datasets other than spinal X-ray or simply underperform. Thus, it is worth exploring replacing the manually designed ensemble rule with a data-driven method. For example, we can introduce an extra neural network which implicitly learns the ensemble rule and merges the segmentation results. This would allow us to avoid manually designing the ensemble rule and make the pipeline more generalized to other domains. (2) Currently the U-Net and Mask R-CNN are trained respectively and independently. This is inefficient and not straightforward compared to an end-to-end approach of training the models together. Training both models together would reduce the computation time while enabling us to dynamically merge their output, as mentioned in the previous point. This would however require further hyperparameter tuning and model designing, beyond the scope of this proof of concept for spinal segmentation. (3) Our proposed pipeline performed well to identify the names for each vertebral body. However, this was achieved by locating a reference vertebral body (i.e., S1 and C2) first and then inferring the rest of vertebral bodies according to their relative position with respect to the reference vertebral body. This means that our pipeline fails if it is unable to detect a reference vertebral body or predicts the wrong location of the reference vertebral body. During the experiments, our pipeline would fail to work in some cases when the reference vertebral body is not detected in X-ray images (e.g. a X-ray scan does not cover the S1 or C2). (4) Some of the vertebral bodies like T1 and T12 are less frequently seen in the X-ray scans compared to others. This may provide our pipeline with fewer samples of these vertebral bodies during the training and the pipeline would underperform on segmenting them. Figure 7 demonstrates the vertebral body distribution over three datasets. We can see that the number of vertebral bodies like T1 and T12 is significantly less than other vertebral bodies which might be a reason that the measures (i.e., Dice and Panoptic Quality) decrease for T1, T11 and T12.

In this study, we proposed an ensemble pipeline that can automatically segment and label vertebral bodies from lateral spinal X-ray images. We demonstrated that our pipeline has outperformed the benchmark models on MEASURE 1 and can generalized well on PREVENT and NHANES II on two measures (i.e., Dice score and Panoptic Quality). In the future, we aim to simplify the proposed pipeline and make the training process end-to-end. Currently, the proposed pipeline requires a reference vertebral body to infer the names of other vertebral bodies. We will also look to make our pipeline less dependent on reference vertebral body detection such that the pipeline can directly predict the labels of each vertebral body.

## Data Availability

Two of the datasets (MEASURE 1 and PREVENT)^36^ used in this study were collected from completed, anonymized clinical trials. Since the X-ray images were anonymized, these are no longer personal data and so no further EC/IRB (Ethics Committees/Institutional Review Board) approvals are required. Another used dataset (NHANES II) is a public dataset that was conducted by the National Center for Health Statistics, and the Centers for Disease Control (NCHS/CDC) during the Second National Health and Nutrition Examination Survey. Further information about this dataset is available at https://www.nlm.nih.gov/databases/download/nhanes.html. The extra labels of reference vertebral bodies for NHANES II can be downloaded at https://zenodo.org/records/10223910.
